# MiR-24-3p as a prognostic indicator for multiple cancers: from a meta-analysis view

**DOI:** 10.1042/BSR20202938

**Published:** 2020-12-02

**Authors:** He Wang, Chunyang Chen, Keke Ding, Weijie Zhang, Jianquan Hou

**Affiliations:** Department of Urology, First Affiliated Hospital of Soochow University, 188 Shizi RD, Suzhou 215006, China

**Keywords:** Clinical characteristics, Human carcinoma, Meta-analysis, MiR-24-3p, Prognosis

## Abstract

A growing number of researches suggest that microRNAs (miRNAs) as oncogene or tumor suppressor genes play a fundamental role in various kinds of cancers. Among them, miR-24-3p, as a star molecule, is widely studied. However, the prognostic value of miR-24-3p is unclear and controversial. We conducted this meta-analysis to evaluate the prognostic value of miR-24-3p in a variety of cancers by integrated existing articles from four databases. PubMed, Embase, Web of Science, and Cochrane Library (last update in March 2020) were searched for approach literature. Hazard ratios (HRs) and odds ratios (ORs) were used to evaluate the association between miR-24-3p expression levels and prognostic value or clinicopathological characteristics, respectively. A total of 15 studies from 14 literature were finally qualified and concluded in the present meta-analysis. A significantly worse overall survival was observed in higher expression of miR-24-3p cancer group for OS (overall survival) of log-rank tests and Cox multivariate regression by fixed effects model. Also, we found a significant correlation between elevated miR-24-3p levels to RFS (recurrence-free survival) and DFS (disease-free survival). In addition, the pooled odds ratios (ORs) showed that evaluated miR-24-3p was also associated with the larger tumor size (≥5 cm) and advanced TNM stage (III and IV). Built on the above findings, elevated expression levels of miR-24-3p may serve as a promising biomarker used to predict the worse prognosis of cancer patients.

## Background

MicroRNAs (miRNAs), a kind of endogenous non-coding RNAs of 18–22 nucleotides in length, negatively regulate target genes expression at post-transcriptional level [1–3]. As either oncogenes or anti-oncogenes, they are found to play vital roles in a wide range of fundamental biological processes, such as proliferation [4,5], differentiation [6,7], apoptosis [8,9], cell cycle [10–13], metastasis [14–16], stress response [17–19], metabolic [20–22] etc. Owing to its detectability and stability in tissues, marrow or blood, a growing number of studies suggest that miRNAs can serve as promising biomarkers for the prognosis of carcinomas [23].

MiR-24-3p (used name was miR-24), a master regulator from the gene cluster of miR-23a–24-27a, has been identified as an onco or oncosuppressor-miR and its expression is closely associated with cancer occurrence and development by recent studies [24,25]. Previous studies showed that miR-24-3p was highly expressed in many carcinomas [26,27]. In addition, the evaluated expression of miR-24-3p was also found to be associated with cancer prognosis and tumor clinicopathological features, but there were some opposite consequences [28,29]. Up to now, a number of studies have been investigated this molecule in many kinds of cancer, but the most individual study has their limits, for example, small sample size or obtaining controversial results, and so on.

Accordingly, to explore the clinical prognostic value of miR-24-3p in various cancers, we performed this systematic review and meta-analysis to give a better understanding.

## Methods

### Literature search strategy

In this meta-analysis, the statement was used as a guideline [30]. We performed a literature search using the online databases, including PubMed, Embase, Web of Science (WOS), and Cochrane Library from inception to March 2020. The terms “miR-24 OR microRNA-24 OR miRNA-24 OR miR24” and “cancer OR tumor OR neoplasm OR carcinoma OR malignancy” were used to determine the correlative literature.

### Inclusion and exclusion criteria

The inclusion criteria were: (1) studies were published in English; (2) miR-24-3p was investigated in carcinomas; (3) studies were identified the correlation between miR-24-3p expression levels and the prognosis of cancer patients; and (4) studies were provided hazard ratio (HR) and its corresponding 95% confidence intervals (CIs) or sufficient data that can further to assess its HR. The exclusion criteria: (1) studies were published in non-English; (2) studies were case report, abstracts, reviews, letters or meta-analysis; (3) studies were not relevant to the prognostic of cancer patients or the prognosis data originated from TCGA; or (4) studies did not offer sufficient data to calculate the HRs and 95% CI.

### Data extraction

Built on the above criteria, all included studies were managed separately by two investigators (H. Wang and C.Y. Chen) and any disagreement were further examined by a third author (K.K. Ding). The following characteristics were collected: the first author's name, year of publication, nationality, cancer type, specimen, method of detection, sample size, type of miRNA, outcome, tumor stage, lymph node metastasis, cut-off value, follow-up time, HR, and its corresponding 95% CI. Moreover, the clinicopathological parameter data were also collected from qualified articles. For studies that not provide HR and 95% CI, the data were extracted from the Kaplan–Meier curves via Engauge Digitizer version 4.1 [31]. The Newcastle–Ottawa Scale (NOS) was used to assess the quality of the pooled studies. High quality required a NOS score ≥ 5.

### Statistical analysis

The present meta-analysis was assessed by Stata SE12.0 software, RevMan5.2 software and Engauge Digitizer 4.1 software. Pooled HRs with their CIs were applied to describe the correlation between the expression of miR-24-3p and relevant survival outcome (OS, DFS, RFS), and the relation between miR-24-3p and relevant clinicopathologic features were also described by pooled odds ratios (ORs) and their CIs. The heterogeneity was evaluated by *I*^2^ statistics and *Q* tests. *P*<0.05 and/or *I*^2^>50% were defined as significant heterogeneity and random effects model was further to used. In addition, a sensitivity analysis was used to evaluate the contribution of each study to the pooled HR and we could further to estimate the stability of the consequence. Finally, we evaluate the potential publication bias by funnel plot, Begg’s test and Egger’s test. *P*<0.05 was known as obvious publication bias [32].

## Results

**Figure 1:**
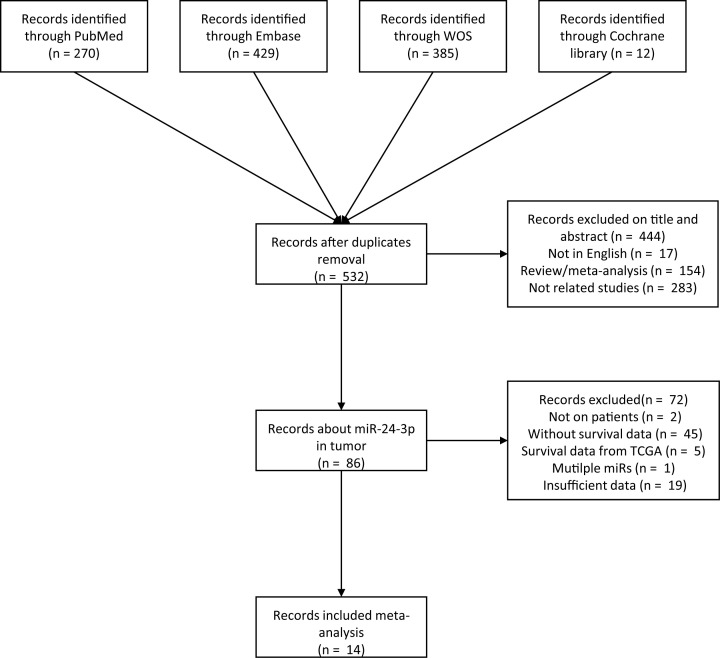
The flow chart of the meta-analysis

As is shown in Figure 1, 1099 literatures were obtained from online databases PubMed, Embase, Web of science (WOS), and Cochrane library. After removing the duplicates, abstract, review, case report, meta-analysis, studies that were not written in English and unrelated researches, 86 articles were subsequently full-text review. Among these, 71 articles were further to removed according to these criteria: studies not on patient (*n*=2), studies without survival data (*n*=45), survival data from TCGA (*n*=5), multiple miRNAs (*n*=1), or insufficient data (*n*=19). Eventually, a total of 15 studies from 14 articles were included (Table 1). The overall sample size is 1518 patients coming from 25 to 247 which from 4 countries. Among these studies, several types of cancer include lung cancer (*n*=3) [33–35], hepatocellular carcinoma (*n*=2) [36,37], colorectal cancer (*n*=2) [29,38], nasopharyngeal carcinoma (*n*=2) [39,40], osteosarcoma (*n*=1) [41], ALL (*n*=1) [42], AML (*n*=1) [42], advanced gastric cancer (*n*=1) [28], esophageal cancer (*n*=1) [43], and head and neck squamous cell carcinoma (*n*=1) [44]. As for OS, RFS, and DFS, there were seven studies directly provide HRs and its 95% CI [28,29,35–39]. In addition, the remaining eight studies only provided Kaplan–Meier curves [28,33,34,40–44]. All studies measured the miR-24-3p expression level by qRT-PCR (quantitative real-time polymerase chain reaction).

**Table 1:** Characteristics of studies included in this meta-analysis

Study (year)	Country	Malignancy	Sample type	Sample (F/M) size (high/low)	Assay	Survival	miRNA	HR (95%CI)	Follow-up	Cut-off value	NOS
Meng et al. (2014)	China	HCC	Blood	72(36/36)	qRT-PCR	OS/DFS	miR-24-3p	OS: 2.364(1.341–4.167) U(Reported)	60	Median	8
								2.141(1.158-3.960) M(Reported)			
								DFS: 2.117(1.197–3.744) U(Reported)			
								2.055(1.114–3.792) M(Reported)			
Meng et al. (2014)	China	CRC	Tissue	95(48/47)	qRT-PCR	OS	miR-24-3p	OS: 0.285(0.139-0.584) U(Reported)	60	Median	8
								0.456(0.212-0.978) M(Reported)			
Kerimis et al. (2017)	Greece	CRC	Tissue	154(115/39)	qRT-PCR	OS/DFS	miR-24-3p	OS: 4.070(1.250–13.19) U(Reported)	120	0.40RQU (the 27th percentile.)	8
								2.600(0.780–8.660) M(Reported)			
								DFS: 4.730(1.120–19.89) U(Reported)			
								4.510 (1.050–19.33) M(Reported)			
Dong et al. (2018)	China	Advanced GC	Tissue	247 (NR)	qRT-PCR	OS	miR-24	OS: 2.945(1.344–4.575) U(Reported)	15	ΔΔCq = -2	9
								3.162 (1.324–4.544) M(Reported)			
Organista-NAVA et al. (2015)	Mexico	ALL	Marrow	111(50/61)	qRT-PCR	OS	miR-24	OS: 2.450(1.500–4.000) U(K-M Curve)	about120	8.22fold(75p)	7
Organista-NAVA et al. (2015)	Mexico	AML	Marrow	36(18/18)	qRT-PCR	OS	miR-25	OS: 1.320(0.030–58.25) U(K-M Curve)	about120	2.54fold(75p)	7
Su et al. (2018)	China	NPC	Tissue	120(60/60)	qRT-PCR	DMFS	miR-24	DMFS: 0.350(0.120–0.980) M(Reported)	about168	Median	6
Wang et al. (2016)	China	NPC	FFPE tissue	25(4/21)	qRT-PCR	RFS	miR-24-3p	RFS: 0.180(0.020–1.660) U(K-M Curve)	about117	–	6
Liu et al. (2014)	China	HCC	Tissue	207(116/91)	qRT-PCR	OS/RFS	miR-24	OS: 2.860(1.650–4.970) U(K-M Curve)	130	–	7
								3.580(2.360–5.460) M(Reported)			
								RFS: 4.280(2.150–8.520) U(K-M Curve)	100		
								4.750(2.660–8.470) M(Reported)			
Zhou et al. (2018)	China	Lung cancer	Tissue	50(25/25)	qRT-PCR	OS	miR-24	OS: 2.392 (0.400–14.45) U(Reported)	80	Median	8
Mori et al. (2016)	Italy	HNSCC	Tissue	108(52/56)	qRT-PCR	RFS	miR-24	RFS: 1.770(1.040–3.800) U(K-M Curve)	about70	Median	6
Yan et al. (2019)	China	Esophageal cancer cancer cancercwdwCancer	Tissue	86(34/52)	qRT-PCR	OS	miR-24	OS: 0.540(0.210–1.350) U(K-M Curve)	20	–	5
Zhao et al. (2015)	China	NSCLC	Tissue	53(39/14)	qRT-PCR	RFS	miR-24-3p	RFS: 1.740(0.390–7.200) U(K-M Curve)	30	Median	8
Liu et al. (2018)	China	Osteosarcoma	Tissue	84(42/42)	qRT-PCR	OS	miR-24	OS: 0.310(0.160–0.630) U(K-M Curve)	50	Median	8
Pan et al. (2018)	China	Lung cancer	Tissue	70(41/29)	qRT-PCR	OS	miR-24	OS: 3.570(1.390–9.150) U(K-M Curve)	60	–	8

Note: The dashes mean no data

Abbreviations: Advanced GC, advanced gastric cancer; ALL, acute lymphocytic leukemia; AML, acute myelocytic leukemia; CRC, colorectal cancer; DFS, disease-free survival; DMFS, distant metastasis-free survival; HCC, hepatocellular carcinoma; HNSCC, head and neck squamous cell carcinoma; M, multivariate; NPC, nasopharyngeal carcinoma; NSCLC, non-small cell lung carcinoma; NOS, Newcastle–Ottawa scale scores; OS, overall survival; RFS, recurrence-free survival; U, univariate.

### The association between miR-24-3p expression levels and the overall survival (OS)

**Figure 2:**
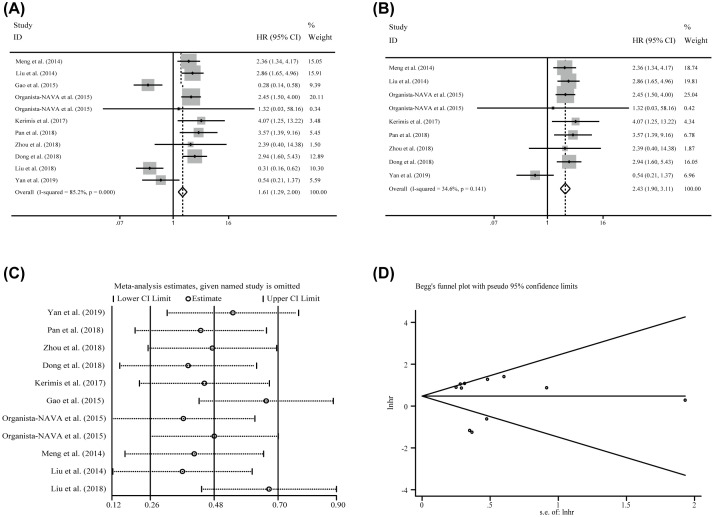
The association between miR-24-3p expression levels and the OS in patients of carcinoma (**A**) overall survival, (**B**) overall survival without the outliers, (**C**) sensitivity analysis, and (**D**) publication bias evaluation.

Ten enrolled articles including eleven studies and 1212 patients were used to investigate the correlation between miR-24-3p expression levels and the OS by using log-rank tests and presented the data of univariate. Generally, a significant correlation between miR-24-3p levels and OS (HR = 1.609, CI: 1.291–2.004, Figure 2A). However, an obvious heterogeneity was also observed (*I*^2^*=*85.20%, *P*≤0.001, Table 2). Hence, the random-effects model was followed in succession but the significance was disappeared (HR = 1.507, CI: 0.810–2.803, Table 2), indicating that the heterogeneity significantly influenced the consequence.

**Table 2:** Association between miR-24-3p expression levels and overall survivals

	Subgroups	No. of studies	No. of patients	Pooled HR (95%CI)		Meta regression	Heterogeneity	
				Fixed	Random	*P*-value	*I*^2^	*P*-value
Overall	OS	11	1212	1.609(1.291–2.004)	1.507(0.810–2.803)		85.20%	≤0.001
Population	Asian (Chinese)	8	911	1.381(1.073–1.777)	1.271(0.585–2.762)	0.341	88.50%	≤0.001
	Non-Asian	3	301	2.615(1.668–4.099)	2.615(1.668–4.099)		0.000%	0.693
Specimen	Tissue	8	993	1.291(0.982–1.698)	1.336(0.574–3.108)	0.505	88.40%	≤0.001
	Non-Tissue	3	219	2.399(1.659–3.470)	2.399(1.659–3.470)		0.000%	0.949
Sample size	≥100	4	719	2.779(2.051–3.766)	2.779(2.051–3.766)	0.069	0.000%	0.873
	<100	7	493	0.882(0.641–1.212)	0.951(0.376–2.404)		85.30%	≤0.001
NOS	≥8	6	700	1.078(0.771–1.507	1.336(0.456–4.094)	0.688	89.60$	≤0.001
	<8	5	512	2.177(1.627–2.913)	1.932(1.152–3.241)		59.80%	0.041
Tumor Category1	Solid tumor	9	1065	1.448(1.131–1.852)	1.427(0.687–2.960)	0.675	87.50%	≤0.001
	Hematologic tumor	2	147	2.425(1.491–3.944)	2.425(1.491–3.944)		0.000%	0.751
Tumor Category2	Digestive system	6	945	1.705(1.291–2.253)	1.520(0.676–3.420)	0.982	87.40%	≤0.001
	Non-Digestive system	5	267	1.461(1.021–2.090)	1.505(0.466–4.863)		85.50%	0.891
Tumor	Esophageal Cancer	1	86	0.540(0.210–1.350)	0.540(0.210–1.350)	—	—	—
	Osteosarcoma	1	84	0.310(0.160–0.630)	0.310(0.160–0.630)		—	—
	Gastric cancer	1	247	2.945(1.344–4.575)	2.945(1.344–4.575)		—	—
	ALL	1	111	2.450(1.500–4.000)	2.450(1.500–4.000)		—	—
	AML	1	36	1.320(0.030–58.25)	1.320(0.030–58.25)		—	—
	Hepatocellular carcinoma	2	279	2.607(1.756–3.871)	2.607(1.756–3.871)		0.000%	0.637
	Lung cancer	2	120	3.274(1.422–7.539)	3.274(1.422–7.539)		0.000%	0.698
	Colorectal cancer	2	249	0.585(0.317–1.080)	1.032(0.076–13.954)		93.00%	≤0.001

Note: The dashes mean no data.

95%CI: 95% confidence interval; Fixed, Fixed effects model; HR, hazard ratio; Random, Random pooling model.

**Figure 3:**
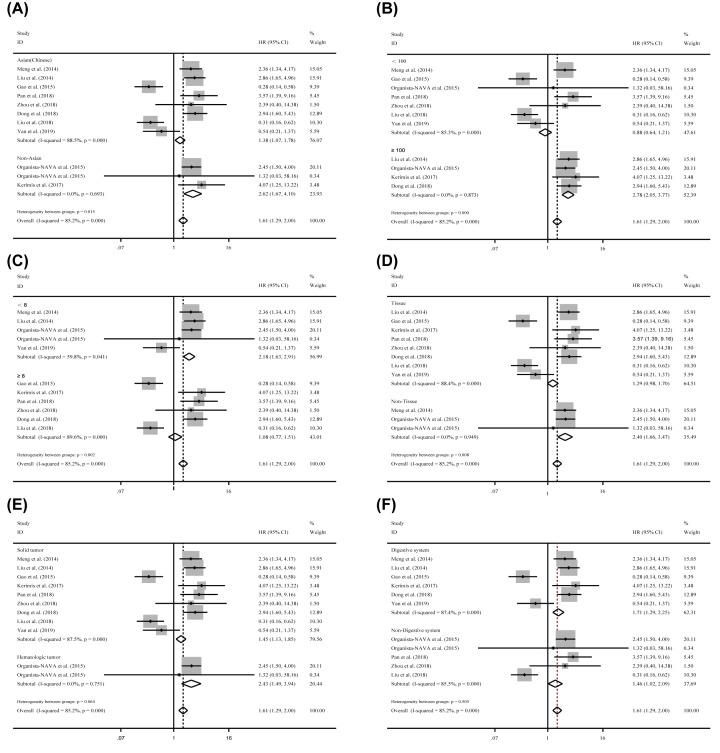
Subgroup analyses for exploring the source of the heterogeneity between miR-24-3p expression levels and the OS (**A**) Population (Asian and Non-Asian), (**B**) sample sizes (<100 and ≥100), (**C**) NOS scores (<8 and ≥8), (**D**) specimen (tissues and non-tissue), (**E**) tumor category1 (solid tumor and hematologic tumor), (**F**) tumor category 2 (digestive system and non-digestive system) for overall survival.

To explore the source of the heterogeneity, the sensitivity analysis was performed, but there was also no positive consequence (Figure 2C). Subsequently, funnel plots, Begg’s test and Egger’s test were implemented to assess the potential publication bias and two studies as the outliers were identified eventually (Figure 2D; Liu et al. [41] and Gao et al. [29]). After removing them, dramatically decline of the heterogeneity was observed (*I*^2^ = 34.60%, *P*=0.141) in the overall analysis, and the significance of the prognostic effects of miR-24-3p expression was still obvious (Figure 2B). Finally, to further explore the source of the heterogeneity, subgroup analyses were applied by factors including population (Asian(Chinese) and Non-Asian), sample size (≥100 and <100), NOS scores (≥8 and <8), specimen (tissue and non-tissue) tumor category 1 (solid tumor and non-solid tumor), tumor category 2 (digestive system and non-digestive system), and tumor (esophageal cancer, osteosarcoma, lung cancer, gastric cancer, colorectal cancer, ALL, AML, and hepatocellular carcinoma) (Table 2). As a consequence, the heterogeneity was controlled successfully in six subgroups and all them have significant correlations: (1) The subgroup of non-Asian (HR = 2.615, CI: 1.668–4.099; *I*^2^ = 0.000%, *P*=0. 693, Figure 3A). (2) The specimen derived from non-tissue (HR = 2.399, CI: 1.659–3.470; *I*^2^ = 0.000%, *P*=0.949, Figure 3D). (3) The sample size greater than or equal to 100 (HR = 2.779, CI:2.051–3.766; *I*^2^ = 0.000%, *P*=0 .873, Figure 3B). (4) The patients of hematologic tumor (HR = 2.425, CI: 1.491–3.944; I^2^ = 0.000%, *P*=0.751, Figure 3E). (5) The patients of hepatocellular carcinoma (HR = 2.607, CI: 1.756–3.871; *I*^2^ = 0.000%, *P*=0.637), and (6) the patients of lung cancer (HR = 3.274, CI: 1.422–7.539, *I*^2^ = 0.000%, *P*=0.698). In addition, significant correlations are also observed in the study of NOS score less than eight by random effects model, which were consistent with the significance of the results by fixed effects model (Table 2). Moreover, significant correlations were observed between miR-24-3p expression levels and OS in the studies with the population derived from Asian (Chinese) (HR = 1.381, CI: 1.219–2.004, Figure 3A), solid tumor (HR = 1.448, CI: 1.131–1.852, Figure 3E), digestive system (HR = 1.705, CI: 1.291–2.253, Figure 3F) and non-digestive system (HR = 1.461, CI: 1.021–2.090, Figure 3F) by fixed effects model, while there were no significances identified in these groups when the random effects model was applied (Table 2). For patients of Colorectal cancer, the prognostic value of miR-24-3p expression levels to the OS was completely different (Kerimis D. et al. [38] HR = 4.070, CI: 1.25-13.19; Gao Y. et al. [29] HR = 0.285, CI: 0.139–0.584). Due to insufficient data, the consequence was lack of efficiency and the heterogeneity was also significant (*I*^2^ = 93.00%, *P*≤0.001). Therefore, more relevant studies are required to perform the analysis. Built on the above consequences, meta-regression was also used, but there was no meaningful contribution identified with impacting on the heterogeneity (Table 2).

### The independent role of miR-24-3p expression levels as a prognostic indicator

**Figure 4:**
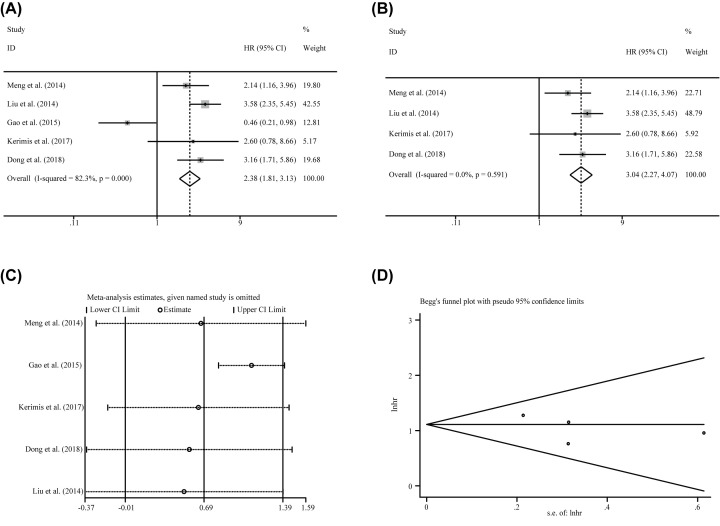
The independent role of miR-24-3p as a prognostic indicator in patients of carcinoma (**A**) overall survival, (**B**) overall survival without outliers, and (**C**) sensitivity analysis, and (**D**) publication bias evaluation.

Five studies containing 775 patients implemented the Cox multivariate regression to assess the prognostic value of miR-24-3p expression levels in carcinoma patients by adjusting other factors. The significant correlation of miR-24-3p expression levels to the OS (HR = 2.384, CI: 1.813–3.134) was observed by fixed effects model. However, the heterogeneity was relatively obvious (*I*^2^ = 82.30%, *P*=0.000, Table 3) and the significance was vanished by random effects model (HR = 1.994, CI: 0.991–4.015). Homoplastically, Subgroup analyses were applied to reduce the heterogeneity. As a result, the homogeneity was reached within the studies of sample size greater than or equal to 100 (*I*^2^ = 0.000%, *P*=0.861), NOS less than 8 (*I*^2^ = 45.50%, *P*=0.176), and the patients of hepatocellular carcinoma (*I*^2^ = 45.50%, *P*=0.176). And the significant association was identified between miR-24-3p expression levels and OS with the sample size greater than 100 (HR = 3.369, CI: 2.414–4.701), NOS less than 8 (HR = 3.041, CI: 2.150–4.300) and the patients of hepatocellular carcinoma (HR = 3.041, CI: 2.150–4.300). In addition, the significant correlations were identified between miR-24-3p expression levels to the OS in the population from Asian(Chinese) (HR = 2.373, CI: 1.813–3.134), the specimen derived from tissue (HR = 2.448, CI: 1.804–3.323) and NOS larger than or equal eight by fixed effects model, which become to no significance within those subgroups by random effects model (Table 3). For patients of Colorectal cancer (HR = 0.752, CI: 0.212–0.978), the prognostic value of miR-24-3p expression levels to the OS was also opposite (Kerimis D. et al. [38] HR = 2.60, CI: 0.780–8.660; Gao Y. et al. [29] HR = 0.456, CI: 0.394–1.434). Thus, more pertinent studies are required to perform the analysis. Similarly, there was no noteworthy contribution identified to greatly influence the variation of HR by meta-regression (Table 3). But the sensitivity analysis suggested that Gao et al. [29] has a significant impact on the result (Figure 4C). The heterogeneity was vanishing (*I*^2^ = 0.000%, *P*=0.591, Figure 4B) by removing this outlier and the correlation of miR-24-3p expression levels to the OS was also significant (HR = 3.039, CI: 2.268–4.074, Figure 4B). Finally, funnel plots, Begg’s test (*P*=0.734) and Egger’s test (*P*=0.460) indicated that there was no bias. But, the number of enrolled studies was few and more data are needed to reinforce this result.

**Table 3:** Meta-analysis of miR-24-3p as an independent prognostic indicator for patients of various carcinomas

	Subgroups	No. of studies	No. of patients	Pooled HR (95%CI)		Meta regression	Heterogeneity	
				Fixed	Random	*P*-value	*I*^2^	*P*-value
Overall	OS	5	775	2.384 (1.813–3.134)	1.994 (0.991–4.015)		82.30%	≤0.001
Population	Asian (Chinese)	4	621	2.373 (1.792–3.142)	1.897 (0.848–4.242)	0.801	86.70%	≤0.001
	Non-Asian	1	154	2.600 (0.780–8.660)	2.600 (0.780–8.660)		—	—
Specimen	Tissue	4	703	2.448 (1.804–3.323)	1.940 (0.765–4.9221)	0.932	86.60%	≤0.001
	Blood	1	72	2.141 (1.158–3.96)	2.141 (1.158–3.960)		—	—
Sample size	≥100	3	608	3.369 (2.414–4.701)	3.369 (2.414–4.701)	0.157	0.000%	0.861
	<100	2	167	1.166 (0.722–1.883)	1.005 (0.221–4.575)		89.50%	0.002
NOS	≥8	3	496	1.593 (1.020–2.488)	1.530 (0.402–5.818)	0.502	87.20%	≤0.001
	<8	2	279	3.041 (2.150–4.300)	2.914 (1.778–4.774)		45.50%	0.176
Tumor	Colorectal cancer	2	249	0.752 (0.394–1.434)	1.021 (0.186–5.594)	—	82.50%	0.017
	Hepatocellular carcinoma	2	279	3.041 (2.150–4.300)	2.914 (1.778–4.774)		45.50%	0.176

Note: The dashes mean no data

95%CI: 95% confidence interval, Fixed: Fixed effects model, HR: hazard ratio, Random: Random pooling model

### The correlation of miR-24-3p expression levels to the RFS /DFS

**Figure 5:**
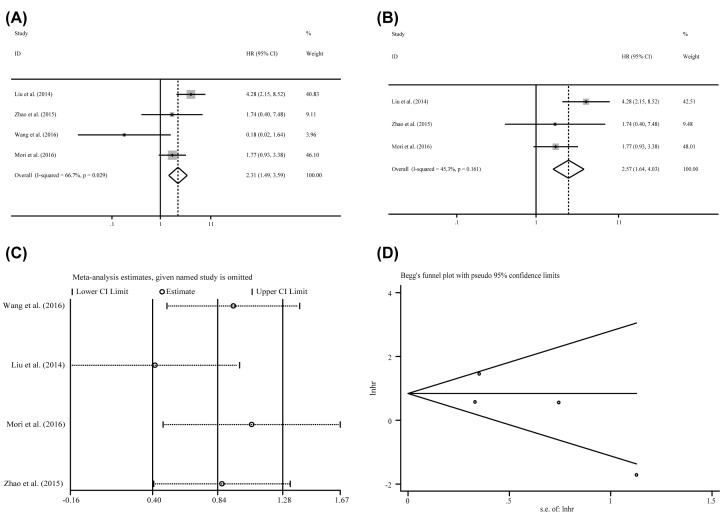
The association between miR-24-3p expression levels and RFS in patients of carcinoma (**A**) Recurrence-free survival, (**B**) recurrence-free survival without the outliers as well as corresponding, (**C**) sensitivity analysis, and (**D**) publication bias evaluation.

Except for OS as a prognostic indicator, RFS and DFS are also be accepted as an evaluation criterion. Here, four studies reported RFS including 393 patients applied log-rank tests, while only one also utilized Cox multivariate regression. After pooling the HR, we observed a significant association between miR-24-3p expression levels to the RFS of log-rank tests (HR = 2.315, CI: 1.491–3.594, Figure 5A) by fixed effects model. However, the heterogeneities were quite obvious (*I*^2^ = 66.70%, *P*=0.290, Table 4). The random effects model was further implemented but the significance was disappeared (HR = 1.814, CI: 0.741–4.440), indicating that the heterogeneity influenced the consequences significantly. Furthermore, owing to the limited number of statistics from Cox multivariate regression, the sensitivity analysis and publication bias were only applied to analysis with data extracted from log-rank tests. The sensitivity analysis result indicated that no studies had significant influences on the consequent (Figure 5C). However, the investigation of potential publication bias identified an outlier (Figure 5D, Wang S. et al. [40]). After deleting this study, the heterogeneity was obvious declined (*I*^2^ = 45. 30%, *P*=0.161) and the significance of the correlation between miR-24-3p expression levels and the RFS was not altered (HR = 2.575, CI: 1.642–4.029, Figure 5B). Due to the limit included studies, more data are needed to enhance the result. In addition, there were only two studies containing 226 patients revealed the DFS statistics and almost no heterogeneity in both log-rank tests and Cox multivariate regression (*I*^2^ = 3.600%, *P*=0.309, *I*^2^=0.000%, *P*=0.330, respectively, Table 4) by used a fixed effects model. We also observed significant strong correlation between miR-24-3p expression levels to the DFS of both log-rank tests (HR = 2.361, CI: 1.390–4.012) and Cox regression tests (HR = 2.313, CI: 1.315–4.067) by fixed effects model.

**Table 4:** Association between miR-24-3p expression levels and other prognostic indicators

		No. of studies	No. of patients	Pooled HR (95%CI)		Heterogeneity	
				Fixed	Random	*I*^2^	*P*-value
RFS	Univariate	4	393	2.315 (1.491–3.594)	1.814 (0.741–4.440)	66.70%	0.029
DFS	Univariate	2	226	2.361 (1.390–4.012)	2.386 (1.362–4.180)	3.60%	0.309
	Multivariate	2	226	2.313 (1.315–4.067)	2.313 (1.315–4.067)	0.000%	0.330

Note: The dashes mean no data

95%CI: 95% confidence interval, Fixed: Fixed effects model, HR: hazard ratio, Random: Random pooling model

### Correlations between miR-24-3p levels and clinicopathological features among various carcinomas

**Figure 6:**
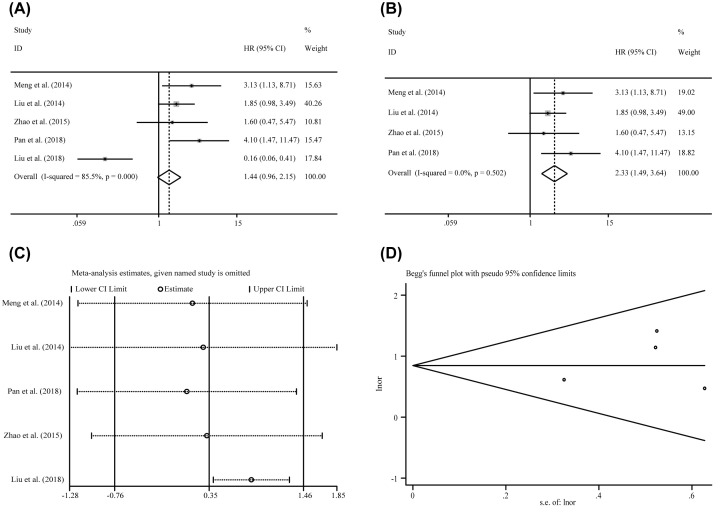
The association between miR-24-3p expression level and TNM stages of cancer patients (**A**) overall pooling result, (**B**) pooling result without the outliers, (**C**) sensitivity analysis, anf (**D**) publication bias evaluation.

Six studies containing 536 patients investigated the correlation of miR-24-3p expression levels to different clinical characteristics. As showed in Table 5, miR-24-3p expression levels were significant correlation with tumor size (OR = 1.655, CI: 1.124–2.437) by the fixed effects model with lesser heterogeneity (*I*^2^ = 37.50%, *P*=0.184). In addition, there were no significance identified in the correlation between age (OR = 0.684, CI: 0.357–1.310), gender (OR = 1.286, CI: 0.758–2.107), lymph node metastasis (OR = 1.591, CI: 0.758–3.339) or TNM stage (OR = 1.437, CI: 0.959–2.154) with the expression levels of miR-24-3p. There was no heterogeneity in the analysis of age (*I*^2^ = 0.000%, *P*=0.525) and gender (*I*^2^ = 0.000%, *P*=0.842), but the heterogeneity of lymph node metastasis and TNM stage were obviously (*I*^2^ = 70.90%, *P*=0.064; *I*^2^=85.50%, *P*=0.000, respectively). To decrease the heterogeneity, sensitivity analysis and publication bias were further investigated to each of them. As a result, an outlier was found (Liu et al. [36]) in the TNM stage. After removing the outlier, the heterogeneity was dramatically decreased from 85.50% to 0.000% and the associations between high miR-24-3p expression levels to advanced TNM stage were significant (OR = 2.328, CI: 1.490-3.637) (Figure 6). Moreover, there was no potential publication bias about TNM stage by funnel plot, Begg’s test (*P*=0.086) and Egger’s test (*P*=0.734). For the analyze of lymph node metastasis, there were only two studies and had an obvious opposite result (Pan et al. [33], OR = 2.974, CI: 1.101–8.037; Zhou et al. [35], OR = 0.725, CI: 0.238–2.208). Due to insufficient data, the consequence would be lack of efficiency and the reasons of heterogeneity were unacceptable. Thus, more pertinent studies are required to perform the analysis.

**Table 5:** Overall analysis of miR-24-3p expression association with clinicopathological characteristics

Clinicopathological parameters	No. of studies	No. of patients	Pooled OR (95%CI)		Heterogeneity	
			Fixed	Random	*I*^2^	*P*-value
Gender	5	329	1.286 (0.758–2.107)	1.286 (0.758–2.107)	0.000%	0.842
Age	2	156	0.684 (0.357–1.310)	0.684 (0.357–1.310)	0.000%	0.525
Tumor size	4	433	1.655 (1.124–2.437)	1.605 (0.985–2.689)	37.50%	0.184
Lymph node metastasis	2	120	1.591 (0.758–3.339)	1.503 (0.377–5.989)	70.90%	0.064
TNM stage	5	486	1.437 (0.959–2.154)	1.419 (0.469–4.291)	85.50%	≤0.001

Note: The dashes mean no data.

95%CI: 95% confidence interval, Fixed: Fixed effects model, HR: hazard ratio, Random: Random pooling model.

## Discussion

It is of great importance to explore prognostic biomarkers with the patients of carcinoma as specific biomarkers can further help to directly stratify patients and effectively guide clinical decision-making. MiR-24-3p, as an oncogene or tumor suppressor, plays a key role in the occurrence, progression and metastasis of human carcinoma was realized by more and more researchers gradually [24,25]. Quan et al. [45] had made a meta-analysis to research the correlation between miR-23a/24-2/27a cluster with human cancers, but they only had limited data to draw a conclusion that high expression levels of miR-23a/24-2/27a indicated a worse prognosis and no further analyzing the correlation between miR-24-3p expression levels to the clinicopathological characteristics. Subsequently, more and more studies that focus on the miR-24-3p expression levels with cancer progression, metastasis and prognosis of patients were carried on [4–7,14–16]. Thus, the exact role of miR-24-3p on the clinical prognosis of patients in various human carcinomas still need to investigate further. In this meta-analysis, total of 15 studies including 1518 people were recruited. Among them, ten studies containing 1212 patients provided the statistics of the OS by log-rank tests. By the pooling strategy, we know that the elevated miR-24-3p expression levels were linked to worse prognosis of cancer patients. Subsequently, several approaches were put in place to investigate the heterogeneity. First, subgroup analyses were conducted to identify the potential sources of heterogeneity. We found that the heterogeneity was achieved within the non-Asian population, non-tissue, the studies of sample size greater than or equal 100, hematologic tumor, hepatocellular carcinoma and lung cancer. But it was not controlled in other groups, such as the Asian population, studies of sample size less than to 100, solid tumor and so on. Second, the sensitivity analysis was used, but no outlier was identified to impact on the pooled results significantly. Third, two studies were identified as outliers by publication bias evaluation (Liu et al. [41] and Gao et al. [29]). After retrieving the data of outliers, we found that the specimen recruited in them were all from Asian (Chinese), tissue and the sample size less than 100 which all have strong heterogeneity. Besides, Liu et al. [41] was the sole study that focused on Osteosarcoma and Gao et al. [29] had an opposite conclusion with Kerimis et al. [38] who also investigated the miR-24-3p expression levels to colorectal cancer. After removing those two outliers, greatly declined of the heterogeneity was observed. Based on the mentioned above, these two studies could be the major sources of heterogeneity. However, more relevant data are needed to further investigate because of the limit number of studies. There were five studies including 775 patients obtained the data of HRs by Cox multivariate regression. Cox multivariate regression has been known as an effective approach because it can evaluate the contribution of each factor independently by adjusting other factors [46]. Thus, the consequences by Cox multivariate regression are always considered as independent effects of each factor on the clinical outcome. As a result, we found that the significance was inconsistent among different effects model. This phenomenon suggested that the heterogeneity was relatively obvious and the consequences were instable. Through the subgroup analyses, we found that the heterogeneity was declined in hepatocellular carcinoma and achieved in the studies of sample size larger than or equal 100. In addition, the sensitivity analysis identified one outlier, Gao et al. [29] who has an opposite conclusion with Kerimis et al. [38]. After removing this study, the heterogeneity had been significantly vanished. High miR-24-3p expression had a significantly worse survival and there was no publication bias. Thus, the power of miR-24-3p expression levels might serve as an independent prognostic indicator and we need more data to reinforce this conclusion. Also, we detected additional indexes, such as RFS and DFS. MiR-24-3p expression levels were deemed to be significantly associated with DFS of statistics extracted from both log-rank tests and Cox regression analysis. For the RFS of cancer patients, only the fixed effects model revealed a significant correlation between miR-24-3p expression with this prognostic index and the heterogeneity was palpable. We identified an outlier (Wang et al. [40]) through publication bias evaluation. After removing the present study, the heterogeneity was declined and the significance of the association between miR-24-3p expression levels and the RFS was not altered.

As for the clinicopathological parameters, six studies including 536 patients had evaluated the association of miR-24-3p expression levels to the distinctive clinical parameters. The overexpression of miR-24-3p was found to be significantly related to larger tumor size by the fixed effects model. Moreover, we found significant heterogeneity between miR-24-3p expression levels to TNM stage. Appling sensitivity analyses, we identified one study (Liu et al. [36]) that had great impact on the result for the TNM stage. After removing this study, the heterogeneity completely disappeared; the association between miR-24-3p expression levels to the TNM stage was also significant. In addition, there were only two studies about the lymph node metastasis are enrolled and the conclusion might be not reliable. The analyzes of clinical features of a definite carcinoma should be normalized for the cut-off values, the feature categories and so on, to enrich the enrolled cases and characteristics for the meta-analysis.

As far as we know, this meta-analysis was the most comprehensive and systematic one to explore the correlation between the miR-24-3p expression levels with the prognosis of cancer patients in depth. Subgroup analysis, meta regression, sensitivity analysis and publication bias had been used to investigate the possible source of the heterogeneity to the greatest extent [47]. In spite of this, several flaws were hard to avoid in this meta-analysis. First, inevitable limitation from insufficient data in this analysis (only 15 studies with 1518 patients). Second, the cut-off values of the miR-24-3p expression levels were not exactly among those studies, thus, the accuracy of prognostic results may be influenced. Third, part of HRs was calculated from the survival curves which may cause some bias. Four, the number of recruited studies for DFS, RFS and clinicopathological features analyses was relatively insufficient. Taking above reasons into account, we need better designed and large sample size studies for further research before applying miR-24-3p as a prognostic biomarker of tumor in clinical applications.

## Conclusions

The overexpression of miR-24-3p was an underlying risk of poor prognosis in various human carcinomas, especially in hepatocellular carcinoma and lung cancer. As for other types of carcinomas, the results are not yet stable and more studies including normalized research conditions are required to identify miR-24-3p prognostic values further. In addition, high miR-24-3p expression levels were linked to the progression of cancers, developing more malignant behaviour, such as larger tumor sizes and the advanced TNM stages. To sum up, miR-24-3p expression levels could serve as a potential prognostic marker of human carcinoma.

## Data Availability

The authors declare that all data supporting the findings of this study are available within the article and the enrolled articles for meta-analysis. The datasets generated and/or analyzed during the current study are available in PubMed, Embase, Web of Science (WOS), and Cochrane library repository. PubMed: https://pubmed.ncbi.nlm.nih.gov/. Embase: https://www.embase.com/login. Web of Science: http://apps.webofknowledge.com. Cochrane Library: https://www.cochranelibrary.com/.

## Abbreviations

95%CI: 95% confidence interval
Advanced GC: advanced gastric cancer
ALL: acute lymphocytic leukemia
AML: acute myelocytic leukemia
CRC: colorectal cancer
DFS: disease-free survival
DMFS: distant metastasis-free survival
Fixed: fixed effects model
HCC: hepatocellular carcinoma
HNSCC: head and neck squamous cell carcinoma
HR: hazard ratio
M: multivariate
NOS: Newcastle–Ottawa scale scores
NPC: nasopharyngeal carcinoma
NSCLC: non-small cell lung carcinoma
OS: overall survival
qRT-PCR: quantitative Real-time PCR
Random: random pooling model
RFS: recurrence-free survival
U: univariate
WOS: Web of Science

## References

[1] Felekkis K., Touvana E., Stefanou C. and Deltas C. (2010) microRNAs: a newly described class of encoded molecules that play a role in health and disease. Hippokratia 14, 236–240 21311629 PMC3031315

[2] Ling H., Fabbri M. and Calin G.A. (2013) MicroRNAs and other non-coding RNAs as targets for anticancer drug development. Nat. Rev. Drug Discov. 12, 847–865 10.1038/nrd4140 24172333 PMC4548803

[3] Liu H., Lei C., He Q., Pan Z., Xiao D. and Tao Y. (2018) Nuclear functions of mammalian MicroRNAs in gene regulation, immunity and cancer. Mol. Cancer 17, 64 10.1186/s12943-018-0765-5 29471827 PMC5822656

[4] Liu M., Zhang Y., Zhang J., Cai H., Zhang C., Yang Z. et al. (2018) MicroRNA-1253 suppresses cell proliferation and invasion of non-small-cell lung carcinoma by targeting WNT5A. Cell Death Dis. 9, 189 10.1038/s41419-017-0218-x 29415994 PMC5833797

[5] Zhuang M., Qiu X., Cheng D., Zhu C. and Chen L. (2018) MicroRNA-524 promotes cell proliferation by down-regulating PTEN expression in osteosarcoma. Cancer Cell Int. 18, 114 10.1186/s12935-018-0612-1 30123092 PMC6090628

[6] Li N., Long B., Han W., Yuan S. and Wang K. (2017) microRNAs: important regulators of stem cells. Stem Cell Res. Ther. 8, 110 10.1186/s13287-017-0551-0 28494789 PMC5426004

[7] Song L. and Tuan R.S. (2006) MicroRNAs and cell differentiation in mammalian development. Birth Defects Res. C Embryo Today 78, 140–149 10.1002/bdrc.20070 16847891

[8] Pileczki V., Cojocneanu-Petric R., Maralani M., Neagoe I.B. and Sandulescu R. (2016) MicroRNAs as regulators of apoptosis mechanisms in cancer. Clujul. Med. 89, 50–55 27004025 10.15386/cjmed-512 PMC4777469

[9] Slattery M.L., Mullany L.E., Sakoda L.C., Wolff R.K., Samowitz W.S. and Herrick J.S. (2018) Dysregulated genes and miRNAs in the apoptosis pathway in colorectal cancer patients. Apoptosis 23, 237–250 10.1007/s10495-018-1451-1 29516317 PMC5856858

[10] Bueno M.J. and Malumbres M. (2011) MicroRNAs and the cell cycle. Biochim. Biophys. Acta 1812, 592–601 10.1016/j.bbadis.2011.02.002 21315819

[11] Mens M. M.J. and Ghanbari M. (2018) Cell Cycle Regulation of Stem Cells by MicroRNAs. Stem Cell Rev. Rep. 14, 309–322 10.1007/s12015-018-9808-y 29541978 PMC5960494

[12] Shirjang S., Mansoori B., Asghari S., Duijf P. H.G., Mohammadi A., Gjerstorff M. et al. (2019) MicroRNAs in cancer cell death pathways: Apoptosis and necroptosis. Free Radic. Biol. Med. 139, 1–15 10.1016/j.freeradbiomed.2019.05.017 31102709

[13] Abdelalim E.M. (2013) Molecular mechanisms controlling the cell cycle in embryonic stem cells. Stem Cell Rev. Rep. 9, 764–773 10.1007/s12015-013-9469-9 23955576

[14] Baranwal S. and Alahari S.K. (2010) miRNA control of tumor cell invasion and metastasis. Int. J. Cancer 126, 1283–1290 19877123 10.1002/ijc.25014 PMC2950784

[15] Kim J., Yao F., Xiao Z., Sun Y. and Ma L. (2018) MicroRNAs and metastasis: small RNAs play big roles. Cancer Metastasis Rev. 37, 5–15 10.1007/s10555-017-9712-y 29234933 PMC5803344

[16] Lou W., Liu J., Gao Y., Zhong G., Chen D., Shen J. et al. (2017) MicroRNAs in cancer metastasis and angiogenesis. Oncotarget 8, 115787–115802 10.18632/oncotarget.23115 29383201 PMC5777813

[17] Du J., Li M., Huang Q., Liu W., Li W.Q., Li Y.J. et al. (2019) The critical role of microRNAs in stress response: Therapeutic prospect and limitation. Pharmacol. Res. 142, 294–302 10.1016/j.phrs.2018.12.007 30553824

[18] Olejniczak M., Kotowska-Zimmer A. and Krzyzosiak W. (2018) Stress-induced changes in miRNA biogenesis and functioning. Cell. Mol. Life Sci. 75, 177–191 10.1007/s00018-017-2591-0 28717872 PMC5756259

[19] Wiegand C., Savelsbergh A. and Heusser P. (2017) MicroRNAs in Psychological Stress Reactions and Their Use as Stress-Associated Biomarkers, Especially in Human Saliva. Biomed Hub. 2, 1–15 10.1159/000481126 31988918 PMC6945927

[20] Dupont C., Kappeler L., Saget S., Grandjean V. and Levy R. (2019) Role of miRNA in the Transmission of Metabolic Diseases Associated With Paternal Diet-Induced Obesity. Front. Genet. 10, 337 10.3389/fgene.2019.00337 31057600 PMC6482346

[21] Huang Y., Yan Y., Xv W., Qian G., Li C., Zou H. et al. (2018) A New Insight into the Roles of MiRNAs in Metabolic Syndrome. Biomed. Res. Int. 2018, 7372636 10.1155/2018/7372636 30648107 PMC6311798

[22] Rottiers V. and Naar A.M. (2012) MicroRNAs in metabolism and metabolic disorders. Nat. Rev. Mol. Cell Biol. 13, 239–250 10.1038/nrm3313 22436747 PMC4021399

[23] Lan H., Lu H., Wang X. and Jin H. (2015) MicroRNAs as potential biomarkers in cancer: opportunities and challenges. Biomed. Res. Int. 2015, 125094 10.1155/2015/125094 25874201 PMC4385606

[24] Kang H., Rho J.G., Kim C., Tak H., Lee H., Ji E. et al. (2017) The miR-24-3p/p130Cas: a novel axis regulating the migration and invasion of cancer cells. Sci. Rep. 7, 44847 10.1038/srep44847 28337997 PMC5364481

[25] Yan L., Ma J., Zhu Y., Zan J., Wang Z., Ling L. et al. (2018) miR-24-3p promotes cell migration and proliferation in lung cancer by targeting SOX7. J. Cell. Biochem. 119, 3989–3998 10.1002/jcb.26553 29231262

[26] Du W.W., Fang L., Li M., Yang X., Liang Y., Peng C. et al. (2013) MicroRNA miR-24 enhances tumor invasion and metastasis by targeting PTPN9 and PTPRF to promote EGF signaling. J. Cell Sci. 126, 1440–1453 10.1242/jcs.118299 23418360

[27] Khodadadi-Jamayran A., Akgol-Oksuz B., Afanasyeva Y., Heguy A., Thompson M., Ray K. et al. (2018) Prognostic role of elevated mir-24-3p in breast cancer and its association with the metastatic process. Oncotarget 9, 12868–12878 10.18632/oncotarget.24403 29560116 PMC5849180

[28] Dong X. and Liu Y. (2018) Expression and significance of miR-24 and miR-101 in patients with advanced gastric cancer. Oncol. Lett. 16, 5769–5774 30405753 10.3892/ol.2018.9324 PMC6202543

[29] Gao Y., Liu Y., Du L., Li J., Qu A., Zhang X. et al. (2015) Down-regulation of miR-24-3p in colorectal cancer is associated with malignant behavior. Med. Oncol. 32, 362 10.1007/s12032-014-0362-4 25502080

[30] Liberati A., Altman D.G., Tetzlaff J., Mulrow C., Gotzsche P.C., Ioannidis J.P. et al. (2009) The PRISMA statement for reporting systematic reviews and meta-analyses of studies that evaluate healthcare interventions: explanation and elaboration. BMJ 339, b2700 10.1136/bmj.b2700 19622552 PMC2714672

[31] Parmar M.K., Torri V. and Stewart L. (1998) Extracting summary statistics to perform meta-analyses of the published literature for survival endpoints. Stat. Med. 17, 2815–2834 10.1002/(SICI)1097-0258(19981230)17:24<2815::AID-SIM110>3.0.CO;2-8 9921604

[32] Irwig L., Macaskill P., Berry G. and Glasziou P. (1998) Bias in meta-analysis detected by a simple, graphical test. Graphical test is itself biased. BMJ 316, 470, author reply 470-471 9492687 PMC2665595

[33] Pan Y., Wang H., Ma D., Ji Z., Luo L., Cao F. et al. (2018) miR24 may be a negative regulator of menin in lung cancer. Oncol. Rep. 39, 2342–2350 29565463 10.3892/or.2018.6327

[34] Zhao G., Liu L., Zhao T., Jin S., Jiang S., Cao S. et al. (2015) Upregulation of miR-24 promotes cell proliferation by targeting NAIF1 in non-small cell lung cancer. Tumour Biol. 36, 3693–3701 10.1007/s13277-014-3008-4 25725584

[35] Zhou N. and Yan H.L. (2018) MiR-24 promotes the proliferation and apoptosis of lung carcinoma via targeting MAPK7. Eur. Rev. Med. Pharmacol. Sci. 22, 6845–6852 30402849 10.26355/eurrev_201810_16153

[36] Liu Y.X., Long X.D., Xi Z.F., Ma Y., Huang X.Y., Yao J.G. et al. (2014) MicroRNA-24 modulates aflatoxin B1-related hepatocellular carcinoma prognosis and tumorigenesis. Biomed. Res. Int. 2014, 482926 24800232 10.1155/2014/482926 PMC3997078

[37] Meng F.L., Wang W. and Jia W.D. (2014) Diagnostic and prognostic significance of serum miR-24-3p in HBV-related hepatocellular carcinoma. Med. Oncol. 31, 177 10.1007/s12032-014-0177-3 25129312

[38] Kerimis D., Kontos C.K., Christodoulou S., Papadopoulos I.N. and Scorilas A. (2017) Elevated expression of miR-24-3p is a potentially adverse prognostic factor in colorectal adenocarcinoma. Clin. Biochem. 50, 285–292 10.1016/j.clinbiochem.2016.11.034 27939727

[39] Su B., Xu T., Bruce J.P., Yip K.W., Zhang N., Huang Z. et al. (2018) hsamiR24 suppresses metastasis in nasopharyngeal carcinoma by regulating the cMyc/epithelialmesenchymal transition axis. Oncol. Rep. 40, 2536–2546 30226609 10.3892/or.2018.6690 PMC6151896

[40] Wang S., Pan Y., Zhang R., Xu T., Wu W., Zhang R. et al. (2016) Hsa-miR-24-3p increases nasopharyngeal carcinoma radiosensitivity by targeting both the 3′UTR and 5′UTR of Jab1/CSN5. Oncogene 35, 6096–6108 10.1038/onc.2016.147 27157611 PMC5102828

[41] Liu L., Pan J., Wang H., Ma Z., Yin J., Yuan F. et al. (2018) von Willebrand factor rescued by miR-24 inhibition facilitates the proliferation and migration of osteosarcoma cells in vitro. Biosci. Rep. 38, 6 10.1042/BSR20180372 PMC6240719 30279208

[42] Organista-Nava J., Gomez-Gomez Y., Illades-Aguiar B., Del Carmen Alarcon-Romero L., Saavedra-Herrera M.V., Rivera-Ramirez A.B. et al. (2015) High miR-24 expression is associated with risk of relapse and poor survival in acute leukemia. Oncol. Rep. 33, 1639–1649 10.3892/or.2015.3787 25672522 PMC4358084

[43] Yan Q., Chen T., Yang H., Yu H., Zheng Y., He T. et al. (2019) The Effect of FERMT1 Regulated by miR-24 on the Growth and Radiation Resistance of Esophageal Cancer. J. Biomed. Nanotechnol. 15, 621–631 10.1166/jbn.2019.2711 31165706

[44] Mori F., Ferraiuolo M., Santoro R., Sacconi A., Goeman F., Pallocca M. et al. (2016) Multitargeting activity of miR-24 inhibits long-term melatonin anticancer effects. Oncotarget 7, 20532–20548 10.18632/oncotarget.7978 26967561 PMC4991473

[45] Quan J., Liu S., Dai K., Jin L., He T., Pan X. et al. (2018) MicroRNA-23a/24-2/27a as a potential diagnostic biomarker for cancer: A systematic review and meta-analysis. Mol. Clin. Oncol. 8, 159–169 29387410 10.3892/mco.2017.1492 PMC5769231

[46] Royston P. and Altman D.G. (2013) External validation of a Cox prognostic model: principles and methods. BMC Med. Res. Methodol. 13, 33 10.1186/1471-2288-13-33 23496923 PMC3667097

[47] Evangelou E. and Ioannidis J.P. (2013) Meta-analysis methods for genome-wide association studies and beyond. Nat. Rev. Genet. 14, 379–389 10.1038/nrg3472 23657481

